# The Costs and Benefits of Animal Experiments: An Evaluation with Bias

**DOI:** 10.3390/ani2010025

**Published:** 2012-01-13

**Authors:** Susanne Prankel

**Affiliations:** Institute of Science and the Environment, University of Worcester, Worcester, WR2 6AJ, UK; E-Mail: s.prankel@worc.ac.uk

The book is part of the Palgrave Macmillan Animal Ethics Series which was developed with the publisher and the Oxford Centre for Animal Ethics. Andrew Knight is a Fellow of the latter. The Centre “aims to demonstrate rigorous intellectual enquiry and the highest standard of scholarship” (p. xv), and the series aims to satisfy the growing appetite for literature in the field. Knight presents a wealth of data on the issue of costs and benefits associated with animal experiments and the book goes beyond its title: it also imparts some information on alternatives. As the author states in the introduction, it represents an extension of his own published work. It is intended as a critical review of the available evidence, an aim which is somewhat different to the meta-analysis claimed by the publisher.

The book is very successful as an organised accumulation of data on animal experimentation, though the interpretation thereof is debatable. The author’s view comes across strongly, which can be disconcerting for a reader looking for a detached evaluation. While the author seems to use the precautionary principle regarding the benefits and costs of animal experiments, his approach to alternatives is less rigorous and credit is given for the mere potential of being beneficial, such that even readers sympathetic to the author’s view will get the impression of bias, which is unfortunate given the care taken over the collection of useful data. Knight discredits animal experiments for failing just one of his very strict criteria for quality. Rightly, he calls for better study design and communication within the scientific community, but flawed study design cannot discredit animal experimentation *per se.* One of Knight’s criteria for quantifying the quality and utility of an animal experiment is related to the number of its quotations in subsequent literature. He is aware of this approach being controversial (p. 46f).

The book contrasts arguments supporting animal experimentation as useful if not necessary with those which suggest they are scientifically counterproductive (as poor models for human conditions) and ethically indefensible. The author concludes that invasive animal experiments, especially, can be misleading and incur costs to animal welfare that cannot be justified. By contrast, supporters of the different guises of utilitarianism find justification for some experiments, even though strong animal rights supporters would not. The book does not focus on ethical debate (the author briefly outlines possible stances in the introduction) but rather on data. A lot of evidence is cited that supports the abolition of animal experiments and questions their utility, e.g., the lack of consistent predictive ability and transferability of data gained from experimentation on animals to human patients. Notably, there is little mention of examples where transferability was good, which one might have expected.

The book covers the use of animals in medical research, toxicity tests and education, particularly championing the abolition of invasive research in primates, such as chimpanzees. Andrew Knight’s book goes well beyond the remit of the title as it includes a chapter on alternatives to animal experiments (including implementation of the three Rs), recommendations and an overview of key regulations governing animal experiments in the EU and US. The section devoted to the use of animals in education is informed by the author’s own challenges against his vet school during his veterinary studies. The space devoted to studies of educational efficacy of humane teaching methods seems peripheral to the title of the book but is useful for the interested reader.

Knight points out that “current regulations governing animal experimentation fall far short of the moral consideration warranted by scientific advances in the understanding of key animal abilities and characteristics” (e.g., from publications such as his own) (p. 3). In such places reference to further literature would have been very useful (e.g., on p. 204 regarding scientific evidence warranting the protection of larval and foetal stages of animals, invertebrates and cephalopods or on p. 49 regarding the utility of chimpanzee models), it would have made the book even more valuable for readers new to the area.

The book is aimed at a varied readership: “all who are interested in the scientific and educational utility of laboratory animal use, and in alternative research, testing, and educational strategies” including scientists, philosophers and undergraduates (p. 6). The format is excellent for a reader that may not wish to peruse the book from cover to cover as it is very well structured with chapters, each containing introductions, descriptive/informative sub-headings and useful summaries, making navigation through the book very easy. Some information is repeated, which means that chapters can be read stand-alone. Chapter 12 summarises the pertinent points of the book and is a condensed version of the individual chapter summaries and a good start for an interested reader. Despite the accumulation of facts and data the book largely reads well and contains a useful list of abbreviations and glossary of key terms. As there are harmonised reporting statistics in the EU, the most useful data collated in the book comes from this area.

The book is a good starting point for a critical reader looking for an introduction to the subject area. It is a good basis for further discussion. It would have been interesting to see an approach where differing views were presented and evaluated (even by two authors, as done in some publications). Here the reader may get the impression that some opposing views are underrepresented.

## Supplementary Files

Supplementary File 1

