# Supplementary material for: The Costs and Benefits of Animal Experiments: An Evaluation with Bias
Source: Animals (Basel). 2012 Jan 13;2(1):25–6. doi: 10.3390/ani2010025 (PMC4494272; doi:10.3390/ani2010025)
Supplement: Supplementary File 1 [file animals-02-00025-s001.pdf]

Animals **2012**, *2*, 85-92; doi:10.3390/ani2010085

OPEN ACCESS

animals

ISSN 2076-2615

www.mdpi.com/journal/animals

Communication

Bias During the Evaluation of Animal Studies?

Andrew Knight

Oxford Centre for Animal Ethics, 91 Iffley Road, Oxford OX4 1EG, UK;
E-Mail: info@AnimalConsultants.org; Tel.: +44-7824-376709

Received: 8 February 2012; in revised form: 22 February 2012 / Accepted: 22 February 2012 / Published: 23 February 2012

Simple Summary: Animal experimentation evokes strong emotional responses in people on both sides of the debate surrounding its ethical status. However, the true level of its usefulness to society may only be discerned by careful examination of reliable scientific evidence. My recent book, *The Costs and Benefits of Animal Experiments*, reviewed more than 500 relevant scientific publications. Recently in this journal, however, a reviewer essentially accused me of bias. Yet the conclusions of my book are based on sound reasoning and strong evidence, and no critic has yet provided any substantive evidence to refute them.

Abstract: My recent book entitled *The Costs and Benefits of Animal Experiments* seeks to answer a key question within animal ethics, namely: *is animal experimentation ethically justifiable?* Or, more precisely, *is it justifiable within the utilitarian cost:benefit framework that fundamentally underpins most regulations governing animal experimentation?* To answer this question I reviewed more than 500 scientific publications describing animal studies, animal welfare impacts, and alternative research, toxicity testing and educational methodologies. To minimise bias I focused primarily on large-scale systematic reviews that had examined the human clinical and toxicological utility of animal studies. Despite this, Dr. Susanne Prankel recently reviewed my book in this journal, essentially accusing me of bias. However, she failed to provide any substantive evidence to refute my conclusions, let alone evidence of similar weight to that on which they are based. Those conclusions are, in fact, firmly based on utilitarian ethical reasoning, informed by scientific evidence of considerable strength, and I believe they are robust.

Keywords: The Costs and Benefits of Animal Experiments; animal experiment; animal study; animal ethics; animal welfare; 3Rs; utilitarian; bias; systematic review; Palgrave Macmillan Animal Ethics Series

---

1. Introduction

Recently in this journal Dr. Susanne Prankel reviewed my new book [1], *The Costs and Benefits of Animal Experiments* [2]. I appreciate the care Dr. Prankel clearly took with several aspects of her review. Unfortunately, however, she appears to have taken objection to the conclusions of my book. In essence, I stated that, "*When considering costs and benefits overall, one cannot reasonably conclude that the benefits accruing to human patients or consumers, or to those motivated by scientific curiosity or profit, exceed the costs incurred by animals subjected to scientific procedures. On the contrary, the evidence indicates that actual human benefit is rarely—if ever—sufficient to justify such costs.*"

2. Accusations of Bias

In her review, however, Dr. Prankel essentially accused me of bias. She stated that my interpretation of the evidence collated within my book was debatable, and that opposing viewpoints were under-represented. She even went so far as to include "*An Evaluation with Bias*" within the title of her review.

This is an unusually bold claim to make within an academic journal, and readers might reasonably assume that it must therefore have been based on evidence and reasoning of corresponding strength. During more than a decade of researching and publishing in this field, on rare occasion I've been similarly challenged by others. Yet neither Dr. Prankel nor any of those others have ever provided any substantive evidence to refute my conclusions, let alone evidence of similar weight to that on which they are based.

Perhaps because it involves procedures such as disease modelling or toxicity testing that can result in particularly high levels of suffering when compared to most other forms of animal use, animal experimentation is especially controversial. Such procedures evoke strong emotional responses in many people, and sometimes, strong criticism, which in turn leads to strong defences by some who support such research. The debate concerning the moral status of such research is thus coloured by strong emotions on either side, and predisposed to polarisation. In such a climate it is easy to stray from sound reasoning and evidence. However, the facts about the human clinical and toxicological utility of animal experimentation may only be discerned by careful examination of reliable scientific evidence, and ethical assessments of such research are only likely to survive critical scrutiny when based on sound reasoning, which is informed by that evidence.

For the record, I am not biased. Or rather, not unduly biased, acknowledging that it is virtually impossible to fully eliminate the myriad biases relating to a multitude of issues that are possessed by all people. In fact, I have gone to considerable lengths to eliminate bias from my book, to the extent possible. My conclusions are firmly based on utilitarian ethical reasoning, which is informed by scientific evidence of considerable strength, and I believe they are robust. Were substantial contrary evidence to emerge, my conclusions would alter accordingly. This presently appears unlikely, however, given the quantity, quality and consistency of the existing evidence on this issue.

3. Utilitarian Ethical Evaluation of Animal Experiments

In essence, my book seeks to answer a moral question of considerable importance to those policy makers responsible for regulating scientific animal use, to society at large, and to the field of animal ethics; namely: *is animal experimentation ethically justifiable?*

As stated in my book: "*The core principle underpinning animal experimentation regulation and policy is that the likely benefits of such research must outweigh its expected costs. Although considerable financial and human collateral costs do exist, the main costs are borne by the animals subjected to such research. And although such research may be directed at yielding benefits for animal species or the environment, the overwhelming majority is intended for human benefit, whether through the advancement of knowledge, through the development or toxicity testing of clinical interventions and consumer or industrial products, or through educational applications.*

This utilitarian cost:benefit analysis underpins all fundamental regulation governing animal experimentation. Directive 2010/63/EU on the protection of animals used for scientific purposes, which directs such animal use in all EU member states, asserts that it is 'essential, both on moral and scientific grounds, to ensure that each use of an animal is carefully evaluated as to the scientific or educational validity, usefulness and relevance of the expected result of that use. The likely harm to the animal should be balanced against the expected benefits of the project.' [3]".

4. Human Clinical and Toxicological Utility of Animal Models

Much contemporary reliance on animal models within biomedical research and toxicity testing is heavily dependent on assumptions of human utility—and, in particular, of reasonable predictivity for human outcomes. In recent years, however, a growing body of systematic reviews have tested these assumptions. In only two of 20 such reviews located during the most comprehensive survey published to date [4,5] did the authors conclude that animal models were either significantly useful in contributing to the development of human clinical interventions, or substantially consistent with clinical outcomes. Furthermore, one of these reviews was contentious. It concluded that rodent models were predictive of the effects of agents aimed at preventing colon cancer recurrence in humans. However, few agents were tested, and two of the three agents tested in mice actually produced different outcomes in humans [6].

Included within these 20 systematic reviews were studies examining the human clinical utility of invasive chimpanzee experiments, of highly cited animal experiments published in leading scientific journals, and of experiments approved by ethics committees at least partly on the basis of specific claims that these animal studies were likely to lead to concrete advances in human healthcare. Seven additional reviews also failed to demonstrate reliable predictivity of human toxicities such as carcinogenicity and teratogenicity. Results in animal models were frequently equivocal, or inconsistent with human outcomes. Chapters 5 and 6 of my book examine these reviews in detail.

Systematic reviews investigating the clinical utility of invasive chimpanzee studies, and the toxicological utility of animal carcinogenicity studies, both form major foci of my book. Such studies are particularly significant, given that other animal models are even less likely to be generally predictive of human outcomes than chimpanzees, and that other fields of toxicity testing are even less

likely to provide public health benefits, than carcinogenicity testing. Given the poor performance of animal models in both of these fields, contemporary reliance on animal models must also be questioned in other fields of clinically oriented biomedical research and human toxicity testing.

5. Factors Limiting Human Utility

A range of factors appear responsible for such poor human clinical and toxicological utility, which are described in detail in Chapter 7 of my book. These include inherent genotypic and phenotypic differences between humans and test animal species; the distortion of animal characteristics such as physiological and immunological parameters, disease predisposition, and cognitive and behavioural characteristics, along with dependent experimental outcomes, by stressful experimental environments and protocols; and the poor methodological quality of many animal studies documented in numerous systematic reviews.

As Dr. Prankel states, "*flawed study design cannot discredit animal experimentation* per se". It is theoretically true that problems arising from stressors and poor methodological quality might be minimised, although fundamental changes to the practice of laboratory animal science would be required, given their widespread prevalence. However, many limitations resulting from interspecies differences are likely to be technically and theoretically impossible to overcome.

6. Costs Incurred by Laboratory Animals

The degrees of actual or likely human utility are major determinants when conducting a utilitarian ethical evaluation of animal experimentation. The other key determinants relate to the nature, severity and prevalence of the impacts experienced by the animal subjects.

Accurate assessment of such impacts is markedly impeded by lack of published national statistics, and by lack of standardisation among countries that do publish them. Nevertheless, it is clear that many millions of animals are subjected to invasive scientific procedures annually. By far the most accurate estimation of global laboratory animal use published to date assessed animal use in 2005. At least 126.9 million non-human vertebrates were subjected to fundamental or medically applied biomedical research, toxicity testing, or educational use; were killed for the provision of experimental tissues or as surplus to requirements; or were used to maintain established genetically modified (GM) strains [7,8]. However, for several reasons this estimate remains highly conservative, and it also excludes certain additional categories raising ethical concerns.

As described in Chapter 4 of my book, a wide variety of stressors have the potential to cause significant stress, fear, and possibly distress in laboratory animals. These may be associated with the capture of wild-sourced species such as primates to supply laboratories or breeding centres; with transportation, which may be prolonged for some animals; with laboratory housing and environments; and with both routine and invasive laboratory procedures.

A substantial minority of all procedures are markedly invasive. These include procedures resulting in death, surgical procedures (excluding minor procedures), major physiological challenges, and the production of GM strains. The best national statistics describing procedural invasiveness originate from Canada. The proportion of markedly invasive procedures there has ranged between approximately 29 and 44 % over the past decade [9] (see also Chapter 3 of my book).

A sizeable majority of all procedures utilise no anaesthetics of any kind. Unfortunately, however, Canadian figures do not indicate anaesthetic use. In this case the best statistics originate from Britain, where procedures conducted without anaesthesia fluctuated between approximately 59 and 69% of annual totals during the past two decades [10] (see also Chapter 3).

To assess animal impacts it would clearly be helpful to know the frequency of analgesic use, the degree of correlation between markedly invasive procedures and anaesthetic or analgesic use, and the prevalence of environmental enrichment and socialisation opportunities. Unfortunately however, such information is reported sporadically if at all, and is excluded from national statistics.

Nevertheless, a large body of studies have demonstrated that the stress caused by laboratory housing and environments, and by relatively common laboratory procedures such as non-invasive handling, *venipuncture* (blood sampling), and orogastric *gavaging* (the insertion of an oesophageal tube to facilitate the toxicity assessment of 'ingested' compounds), may all result in profound, statistically significant distortions in a range of physiological indices, including cardiovascular parameters and serum concentrations of glucose and various hormones. Behaviour may be markedly altered, and behavioural stereotypies and increased aggression may develop over time, as may alterations in certain neuroanatomical parameters and even cognitive capacities [11–13] (see also Chapter 4). Some of these effects are also likely to be sequelae of other stressors, such as invasive procedures and transportation.

Such stressors do not only create significant animal welfare and ethical problems. As previously noted, the resultant effects on laboratory animals may distort a range of experimental outcomes, such as those dependent on accurate determination of physiological or behavioural characteristics.

7. A Debatable Interpretation?

This evidence describing the human clinical and toxicological utility of invasive animal research, and the impacts experienced by laboratory animals, is drawn from more than 500 scientific publications cited within my book. Although certain knowledge gaps remain, I believe it is nevertheless clear that sufficient evidence now exists to support the conclusion that the human benefits accruing from invasive animal research do not generally exceed the costs incurred by the animals subjected to that research. Dr. Prankel, however, apparently found this conclusion debatable, accusing me of under-representing opposing viewpoints.

As briefly acknowledged in Chapter 12 of my book, a diverse range of philosophical, cultural and religious viewpoints about our moral duties towards animals and people could be applied to scientific animal use. Examination of these viewpoints was not the purpose of my book, however, which was primarily concerned with a detailed examination of the published scientific evidence describing the human benefits of such research, and the costs incurred by the animals subjected to it.

If my main conclusion is wrong, then it means that the human benefits of such research *do* generally exceed the costs incurred by the animals. The most important corollary would be that such research *is* normally justified within the utilitarian philosophical framework most commonly applied to the regulation of animal experiments.

However, I believe it is only possible to draw such a conclusion if a profoundly unequal weighting is applied, in which relatively infrequent or minor human benefits are considered more important than the significant adverse impacts commonly experienced by literally millions of laboratory animals.

Yet such a weighting of interests is increasingly inconsistent with our growing understanding of the psychological and social characteristics of the species used in laboratories, including their ability to experience suffering and pleasure; of the impacts that result from laboratory environments and even relatively common laboratory procedures; and of the moral implications that stem from this knowledge.

8. The Evaluation of Evidence

Dr. Prankel provided several additional criticisms relating to the evidence contained in my book, and its evaluation. She criticized me for making little mention of examples where transferability of data gained from animal experimentation to human patients was good. Such examples do exist, as do counter-examples in which transferability of data was poor. Yet, I deliberately chose not dwell on either, because, "*only small numbers of experiments are normally reviewed in [such] case studies, and their selection may be subject to bias.*" (Chapter 5). Instead, I relied primarily on large-scale systematic reviews as my primary form of evidence. As stated, "*To provide more definitive conclusions, systematic reviews of the human clinical or toxicological utility of large numbers of animal experiments are necessary. Experiments included in such reviews should be selected without bias, via randomisation or similarly methodical and impartial means.*"

An example of such a systematic review was that published by Hackam & Redelmeier [14], which I described in Chapter 5. This study examined the transferability of the tiny proportion of animal studies that were published from 1980–2000 in the seven leading scientific journals when ranked by citation impact factor, that had received more than 500 citations each. The authors reasonably assumed that such animal studies would be most likely to translate to human clinical trials. Of 76 animal studies meeting these criteria, they found that 36.8% (28/76) were replicated in randomised human trials, 18.4% (14/76) were contradicted by such trials, and 44.7% (34/76) had not translated to clinical trials by May 2006. Such failure to translate often indicates that concerns have arisen about human safety or efficacy.

Some of the other systematic reviews I described used citation analyses to evaluate the importance of animal studies in contributing to future publications, particularly those describing clinical interventions ultimately efficacious in humans. As Dr. Prankel noted, the use of citation analyses is controversial. As stated in Chapter 5 however, "*Citation frequencies are not, of course, a definitive indication of the benefits, or lack thereof, of scientific research. Uncited studies may also contribute to the advancement of biomedical knowledge, through a variety of mechanisms. However, citation frequencies do generally provide a quantifiable and reasonably objective approximation of utility, or lack thereof, and they have been shown to be among the most reliable indicators of research quality* [15–17]. *Research that makes a significant contribution to a field—such as by confirming or refuting hypotheses—is very likely to be cited by future papers, as is research that produces interesting or controversial outcomes. On the other hand, research that is inconclusive or of little interest or significance is much less likely to be cited.*" The fact that a sizeable proportion of the animal studies

examined were not subsequently cited by publications of any kind, let alone by human clinical publications, is therefore an issue of legitimate, and considerable, concern.

Finally, my book also comprehensively reviewed the use of animals within life and health sciences education, and alternative research, toxicity testing and educational strategies. With respect to such alternatives, Dr. Prankel criticized me for giving credit "*for the mere potential of being beneficial*", whilst denying similar credit to invasive animal experiments. This claim is itself debatable, however. As I concluded in Chapter 8, "*Non-animal investigative methods cannot, of course, provide answers to all questions about humans, particularly given present technological limitations. However, the same is certainly true of animal models, which have a more limited capacity for further development.*" Such an evaluation is hardly one-sided. Yet, different standards are actually warranted when ethically evaluating the use of animal and non-animal models, because large numbers of animals are seriously harmed and killed in animal studies, but not when non-animal models are used. Accordingly, the benefit of the former must be established far more strongly in order to achieve an equivalent ethical status—a point not acknowledge by Dr. Prankel.

9. The Palgrave Macmillan Animal Ethics Series

Finally, the International Standard Book Numbers (ISBNs) provided by Dr. Prankel were not actually those of my book as stated, but were for the Palgrave Macmillan Animal Ethics Series [18]. Produced in partnership with the Ferrater Mora Oxford Centre for Animal Ethics, the series aims to provide key introductory and advanced texts that map out ethical positions on animal issues, and that are interdisciplinary in nature. At the time of writing six of fifteen books commissioned within the series had been published, of which my book was the third. The correct ISBN for my book, which is available in hardback from Palgrave Macmillan, is 978-0-230-24392-7.

Conflict of Interest

I am the author of *The Costs and Benefits of Animal Experiments* [2], which was the subject of Dr. Susanne Prankel's review [1].

References and Notes

1. Prankel, S. The costs and benefits of animal experiments: An evaluation with bias. *Animals* **2012**, *2*, 25–26. Available online: http://www.mdpi.com/2076-2615/2/1/25/ (accessed on 22 February 2012).
2. Knight, A. *The Costs and Benefits of Animal Experiments*; Palgrave Macmillan: Basingstoke, UK, 2011.
3. European Union. Directive 2010/63/EU of the European Parliament and of the Council of 22 September 2010 on the protection of animals used for scientific purposes. *Official J. EU* **2010**, *276*, 33–79.
4. Knight, A. Systematic reviews of animal experiments demonstrate poor human clinical and toxicological utility. *Altern. Lab. Anim.* **2007**, *35*, 641–659.

5. Knight, A. Systematic reviews of animal experiments demonstrate poor contributions toward human healthcare. *Rev. Recent Clin. Trials* **2008**, *3*, 89–96. Available online: http://www.bentham-direct.org/pages/content.php?RRCT/2008/00000003/00000002/0002RRCT.sgm (accessed on 22 February 2012).
6. Corpet, D.E.; Pierre, F. How good are rodent models of carcinogenesis in predicting efficacy in humans? A systematic review and meta-analysis of colon chemoprevention in rats, mice and men. *Eur. J. Cancer* **2005**, *41*, 1911–1922.
7. Taylor, K.; Gordon, N.; Langley, G.; Higgins, W. Estimates for worldwide laboratory animal use in 2005. *Altern. Lab. Anim.* **2008**, *36*, 327–342.
8. Knight, A. 127 million non-human vertebrates used worldwide for scientific purposes in 2005. *Altern. Lab. Anim.* **2008**, *36*, 494–496.
9. Canadian Council on Animal Care (CCAC). *2008 CCAC Survey of Animal Use*; CCAC: Ottawa, ON, Canada, 2009. Available online: http://ccac.ca/Documents/Publications/Statistics/ Survey_2008.pdf (accessed on 22 February 2012).
10. Home Office. *Statistics of Scientific Procedures on Living Animals: Great Britain 2009*; The Stationery Office: London, UK, 2010. Available online: www.homeoffice.gov.uk/rds/ scientific1.html (accessed on 22 February 2012).
11. Balcombe, J.; Barnard, N.; Sandusky, C. Laboratory routines cause animal stress. *Contemp. Top. Lab. Anim. Sci.* **2004**, *43*, 42–51.
12. Balcombe, J. Laboratory environments and rodents' behavioural needs: A review. *Lab. Anim.* **2006**, *40*, 217–235.
13. Baldwin, A.; Bekoff, M. Too stressed to work. *New Scient.* **2007**, *194*. Available online: http://dx.doi.org/10.1016/S0262-4079(07)61358-X (accessed on 22 February 2012).
14. Hackam, D.G.; Redelmeier, D.A. Translation of research evidence from animals to humans. *J. Am. Med. Assoc.* **2006**, *296*, 1731–1732.
15. Callaham, M.; Wears, R.L.; Weber, E. Journal prestige, publication bias, and other characteristics associated with citation of published studies in peer-reviewed journals. *J. Am. Med. Assoc.* **2002**, *287*, 2847–2850.
16. Lee, K.P.; Schotland, M.; Bacchetti, P.; Bero, L.A. Association of journal quality indicators with methodological quality of clinical research articles. *J. Am. Med. Assoc.* **2002**, *287*, 2805–2808.
17. Rochon, P.A.; Gurwitz, J.H.; Cheung, C.M.; Hayes, J.A.; Chalmers, T.C. Evaluating the quality of articles published in journal supplements compared with the quality of those published in the parent journal. *J. Am. Med. Assoc.* **1994**, *272*, 108–113.
18. Further information about the Palgrave Macmillan Animal Ethics Series is available online: http://www.palgrave.com/products/series.aspx?s=pmaes (accessed on 22 February 2012).

© 2012 by the authors; licensee MDPI, Basel, Switzerland. This article is an open access article distributed under the terms and conditions of the Creative Commons Attribution license (http://creativecommons.org/licenses/by/3.0/).
